# Survivors of childhood cancer in Latin America: Role of foundations and peer groups in the lack of transition processes to adult long‐term follow‐up

**DOI:** 10.1002/cnr2.1474

**Published:** 2021-06-16

**Authors:** Nuria Rossell, María Fernanda Olarte‐Sierra, Julia Challinor

**Affiliations:** ^1^ Independent Medical Anthropology Researcher San Salvador El Salvador; ^2^ Independent Medical Anthropology Researcher Bogotá Colombia; ^3^ University of California San Francisco California USA

**Keywords:** childhood cancer, foundations, Latin America, qualitative research, survivors, transition

## Abstract

**Background:**

Over the last decade, the population of childhood cancer survivors has rapidly increased in Latin America, opening a long chapter of challenges for healthcare providers in these countries to provide follow‐up and adult care.

**Aim:**

In the process of exploring childhood cancer parent and patient engagement in resource‐limited settings, we highlight the challenges faced by Latin American survivors from El Salvador, Mexico, and Peru as they transitioned from receiving cancer treatment to life as a cancer survivors.

**Methods and Results:**

Focus group discussions and interviews were performed as part of a larger qualitative study involving 10 low and middle‐income countries in four continents regarding patient and caregiver engagement in childhood cancer treatment. We present the results of the Latin‐American survivors and their experiences finishing treatment and life outside the pediatric oncology follow‐up system. Themes regarding a) losing eligibility for pediatric surveillance and care, b) the importance of peer survivors, and c) the need for giving back were part of their stories.

**Conclusion:**

We suggest that given the lack of organized support from healthcare systems and providers for survivors' proper transition into adult‐centered care, foundations and non‐governmental organizations can provide transitional support, offer space for guidance/information, and work towards collaboration among systems for future integrated programs.

## 1 INTRODUCTION

Incidence rates per million person‐years for childhood cancer (0–14 years) in Latin America range from 128.1 in Argentina to 153.1 in Peru.^1^ Current rates of survival lag behind high‐income countries, for example, acute lymphoblastic leukemia (ALL) 5‐year survival is 64.5% across Latin America compared to 89% in Western Europe.^2^ This reflects significant progress made over the last few decades in improved access to care and increased resources for childhood cancer treatment. Strategies include twinning partnerships with high‐income countries and galvanizing local non‐governmental organization (NGO) support (e.g., El Salvador), childhood cancer healthcare policy changes (e.g., Mexico),^3^ international NGO projects (e.g., Sanofi Espoir Foundation “My Child Matters” award),^4^ and the WHO Global Initiative for Childhood Cancer focus country status (e.g., Peru).^5^

In 2019, a study was conducted by the International Society of Paediatric Oncology (SIOP), Pediatric Oncology in Developing Countries Committee (PODC), Patient Family and Stakeholder Engagement Task Force (PFSE Task Force) to determine the extent of patient and caregiver engagement during treatment. Ten low‐ and middle‐income countries (LMICs) participated from across 5/6 WHO regions. This paper reports on the results of three of these sites, Peru, El Salvador, and Mexico.

We highlight the challenges faced by Latin American survivors from these three countries as they transitioned from receiving cancer treatment to life as a cancer survivor. We argue that in Latin America, such a transition occurs without coordinated multidisciplinary support from healthcare services and providers; thus, former patients must navigate both their health and life reintegration beyond cancer treatment. Survivors are faced with the task to imagine and live a life as cancer survivors for which they are not necessarily prepared. They find ways to make sense of their new status in multiple ways. Here we address three themes from our respondent interviews.

Based on the survivors' testimonies and stories, we suggest leveraging and/or promoting the involvement of NGOs and childhood cancer foundations that can offer strategies to minimize the abrupt disruption in care as children/adolescents move from cancer treatment to survivor surveillance, adult healthcare and life beyond cancer treatment.

## 2 METHODS

### 2.1 Settings

The detailed methods of the larger study are available elsewhere.^6^ In brief, in El Salvador, the local foundation Ayudame a Vivir (FAV), supporting psychosocial care and >50% of the cost of childhood cancer at the only pediatric hospital in the country, served as entry point to contact participants. In southwest Mexico, the local foundation, Canica, providing psychosocial support to families of children with cancer, contacted potential respondents. In Peru, two local pediatric oncologists (one on temporary leave) recruited survivors and parents from two hospitals to participate in the study. In El Salvador, a group of survivors function under the guidance of a psychologist from FAV and holds monthly meetings and visits to the pediatric oncology ward to talk with parents and children, many of them newly diagnosed. In Mexico, a group of survivors collaborates with Canica activities, participating in camps for children with cancer, and also started a theater play based on their treatment experiences. Some, but not all, the participants in this study are part of these groups. In Peru, there are no organized activities for survivors. The maximum age for admission in the hospital in El Salvador is <12 years old; in Mexico <16, Peru <15 in one hospital, and <18 in the second one.

### 2.2 Post‐cancer treatment standard care

Information about common practices for childhood cancer end of treatment in our Mexican site was not available, however, for the two other countries, it is as follows:

In El Salvador, patients who finish treatment receive 10‐years of follow‐up or until they reach 18 years of age with their pediatric treating team. Monthly check‐up appointments are programmed initially and gradually distanced depending on the child's diagnosis and age, and sometimes particular treatment circumstances. Children who reach the age limit for hospital admission (12 years) during treatment, are not affected by this rule and receive treatment until completion and through follow‐up. Telemedicine follow‐up with calls and test results were initiated this year, especially for those without detected medical conditions or who had reached the last years of long‐term follow‐up. At the time of discharge when after‐treatment follow‐up ends, all survivors are advised to register in the national social security health system. Survivors with conditions that require treatment by other specialists are referred to the adult care system. Currently, there's no integrated system in place; the protocol for follow‐up and referral is not formalized, and there's no follow‐up to determine how the survivors proceed once discharged from the pediatric system.

In one of the hospitals in Peru, follow‐up occurs for at least 5 years, and the same teenager‐treatment team continues follow‐up even after the survivors reach the hospital admission age limit (18 years). In the second hospital, after‐treatment follow‐up continues until the children reach the hospital admission age limit (15), then they have to move to adult services for treatment or follow‐up.

### 2.3 Participants, data collection, and analysis

An El Salvadorean pediatric oncology psychologist/medical anthropologist and a Colombian medical anthropologist conducted all research. Appropriate permission to conduct the studies was arranged according to local practice in each country. In‐depth interviews and focus‐group discussions were conducted with adolescents and young adults who had finished cancer treatment at least 5 years prior.

A Spanish‐language interview and focus group discussion (FGD) guide was developed based on the researchers' clinical experience and literature review about patient engagement, together with expert pediatric oncology professionals and parents of children who had had cancer, all of whom were members of the extended PFSE Task Force research group. Topics included: individual and family experiences during and after treatment regarding information and communication, support systems or strategies, decision‐making, and everyday life in the hospital and at home. Interviews and FGDs were recorded with participants' oral authorization. Transcriptions and content analysis were performed manually and independently by the two researchers. Consensus on the main themes was reached in discussion meetings among the three authors.

## 3 RESULTS

We interviewed 30 survivors from February to September in 2019. (Mexico = 9; El Salvador = 9 and Peru = 12). The ages were from 13 to 31 years and their diagnoses were acute lymphoblastic leukemia, lymphoma, neuroblastoma, bone tumor, and germ cell tumor. Themes that emerged on survivorship included: a) losing eligibility for pediatric surveillance and care, b) the importance of peer survivors, and c) the need for giving back.

### 3.1 Losing eligibility for pediatric surveillance and care

In the three countries, once treatment is completed, survivors have a routine period of a few years (depending on age and diagnosis) of follow‐up appointments with their pediatric oncologists. However, once this period is complete, they are officially discharged from pediatric oncology care and no further appointments are scheduled. To our knowledge, there was no system in place for referral of survivors of childhood cancer to the adult healthcare system.

However, some of our young adult survivors recalled recommendations from their pediatric oncologists during their follow‐up appointments about avoiding alcohol and cigarette consumption and having a healthy, responsible, lifestyle. Nevertheless, most of the respondents who had been teenagers at the time of diagnosis, regardless of their age at the end of treatment, recalled receiving little or no information about survivorship, long‐term treatment effects, or other important topics for their physical and mental health as a childhood cancer survivor (e.g., risk of infertility, second malignancy, cardiac problems, depression, and stigma). A few survivors we spoke with mentioned asking questions to their pediatric oncologists during their follow‐up appointments mainly about lifestyle and allowed activities as they entered young adulthood. They mentioned being very happy about finishing their treatment, mainly because it meant the end of the unpleasant experience, but also because it gave them a sense of relief and having accomplished “the end of the race.” For some, it meant the return to their normal life, which was highly anticipated. For example, Erika was 10 when she was diagnosed with acute lymphoblastic leukemia (ALL). She and her parents moved from their hometown (a 24‐h bus ride) to a small rented apartment close to the hospital in Lima, Peru's capital city during her 2 years treatment. After she was able to return home following completion of her treatment, Erika felt that she could continue with her life with reasonable normality:

… then everything finished: the chemotherapy, the pills, the whole treatment ended. It was a success. The doctor told me that everything went well, and that I could continue with my normal life, but without excesses. And that's it. We went back to cnr21474 [home] until I turned the age of majority, 18 years old, and I moved by myself back to the capital city to study; I lived with a roommate. My father paid [the rent] monthly, paid the university, and everything was fine… (Erika, 22, Survivor of ALL)

In this sense, some respondents reflected that when they had finished treatment, they did not have questions about their future or the long‐term treatment consequences, they were too young for such worries, both during and after treatment. It was only after they entered young adulthood that questions about their treatment and late effects arose as their lifestyles and interests changed due to pending adulthood. The following quotations serve as an example of worries of a young adult cancer survivor:

There are things that I did not ask at the moment because I was not interested, maybe now I'm interested and I'd like to know. I think it depends on the phase [age] when your treatment is happening to have certain questions. Mostly will be ‘why am I losing my hair?’, but asking *grown‐up* questions is more complicated. (Ana, 26, survivor of ALL)

It is rather now that doubts [questions] are coming up for me. For example, I got an ovary removed [during treatment], maybe it is because I'm growing up, or I don't know, but now I'm interested in knowing what happens with my body, whether I'll get to have children; and maybe back at that time it didn't worry me because I was a child; it is rather now that doubts start to come about how much impact the treatment I received got on my body. (Lina, 20, survivor of a germ cell tumor)

We found that, in general, and given the absence of a referral system that supports the transition from childhood care to adult services, survivors in this study trusted their pediatric oncologists as their primary source of follow‐up medical consultation. This was the case both for minor issues after ending their cancer treatment as well as for the follow‐up period, which supposed that they did not use adult healthcare services. Furthermore, survivors reported that they consulted their trusted pediatric oncologist for random ailments and not necessarily for post‐treatment medical conditions requiring a specific follow‐up. Ivan, a survivor of ALL, mentioned:

Those with whom I had closer relationships were my doctors, more than any other staff; mainly with Dr. XX, who was the last one who treated me. It was a very close relationship because we saw each other daily… I could share my doubts [questions], knowing that they would answer them, I felt total trust with them… There was only one doctor, I don't remember her name, but she was very rough, very direct and cold, I never had a good relationship with her. Otherwise, I managed to have a good relationship with all the doctors, and for example, with Dr. XX, even now, when suddenly arises a little worry or something comes up, I can call him without… ‘listen Dr, so and so happened’, and he gives me an appointment immediately… (Ivan, 21, survivor of ALL)

Ingrid finished treatment for ALL 12 years ago; she arranges for routine blood tests and takes the results to her pediatric oncologist.

I went to the hospital to show my results; I run my tests, I don't know, every six months, sometimes yearly, just to keep checking, or, if I feel unwell, I do tests; but if I don't feel ill, only every six months or yearly. So, I did the tests and took them to doctor XX [her pediatric oncologist]. Everything was fine, and he asked me whether I had any other problem or something, and I get low [blood] pressure frequently, it is not that I have that problem [meaning it's not a physiological condition or a disease], it is due to anxiety… so he said ‘Do you want us to help you?’ He asked my authorization to help me; I had scratches, which was a way to release anxiety, and then is when I said [to myself], ‘I can't continue like this’, I reached the limit, and then [is when] I started to go to psychological therapy. (Ingrid, 20, survivor of ALL)

Here it is important to note that many survivors have developed a good relationship with their pediatric oncologists during treatment, which was an essential pre‐condition for further off‐treatment contact with healthcare providers. This situation raises questions about what happens to survivors who, for reasons beyond the scope of this research, are not able to establish meaningful relationships with their pediatric oncology healthcare team or adult healthcare providers.

### 3.2 Importance of peer survivors

In Mexico and El Salvador, local childhood cancer foundations conduct meetings and activities with childhood cancer survivors who have finished their follow‐up appointments. This allows them to sporadically keep in touch with their pediatric healthcare team, other survivors, and children still on treatment and their parents. In Peru, we did not see such engagement with survivors. This might have been due to the lack of an established NGO for childhood cancer engaged in survivors activities.

Despite the efforts made by local cancer foundations, like the ones included in this study, most of our young adult respondents had not met childhood cancer survivors during their treatment. The absence of actual living referents of childhood cancer survivors made the possibility of cure only an ideal without visible proof. This situation further strengthened survivors' feelings of being alone in the face of what was felt as unknown territory. Ana, a survivor of ALL expressed her experience:

There was no survivors' group, that would tell you, ‘okay, you will live, you can make it, I went through this…’ you would only hear ‘xx was taken to the ICU, and never came back’. You would not meet those who made it. (Ana, 26, survivor of ALL)

Thus, having the opportunity to share with peer survivors after treatment offered survivors the possibility to see themselves as triumphant, recognize the great achievement that is surviving childhood cancer, and especially, not feeling alone and isolated since they can see their experience reflected in one another. Some of the participants of this study have organized visits in small groups to the oncology ward where they had received treatment themselves. During these visits, they tell their story as patients and survivors to the parents and children in the ward. However, this is not an experience that is equally easy for all. Ana told us that even now, after more than 15 years of having finished treatment, her mother cannot understand why Ana likes to remember her experiences. Ana recognizes that at the beginning it was difficult to tell her story, but now she feels it is a healing and strengthening experience.

Even today when I told her [my mother] that I was coming [to the hospital], she said: ‘I don't know how you do to remember every day what happened to you, I don't understand where you get that strength from’; and I tell her, ‘for me this is catharsis; when something is hard, or a difficulty, you have to do it at some point. I get unburden by telling [my story] to someone else…’ Initially it was difficult, I'd get a lump in my throat, now I'm super chilled, like: (with excited gesture) ‘look, I'm going to tell you my story, I once had cancer…’ I like that experience [of telling the story] because it gives to the other one, maybe, more courage, more strength for what it's going through. (Ana, 26, survivor of ALL)

Regular contact with other survivors enhanced our respondents' sense of belonging and brother/sisterhood and helped them to share worries, explanations, and answers regarding their common past and uncertain future. They recognize that the bond among survivors is a strong one and they have developed meaningful friendships since they are with those who know and understand like no one else what they have been through, no matter the type of cancer or treatment. This shared experience creates a sense of belonging among childhood cancer survivors.

We also found that gathering with other survivors is an opportunity to compare their questions about long‐term consequences of treatment and determine whether small ailments or discomforts are shared by others or if they are individual experiences. For example, Lina has checked with her friends about little physical annoyances, from gastric burning to joint‐cracking, or mouth blisters, and concluded that everything must be related to long‐term effects of treatment that not only she has experienced.

… the other day talking with some of my friends who are also survivors I noticed that I was not the only one [with tachycardia], which was strange because it is something that the doctors never talked about, for example, I feel that my bones got damaged because I get tired more easily, my knees crack, or little things like that… I notice that since I finished chemotherapy, I get mouth blisters more often… sometimes talking among my friends who are also survivors, they also tell me what is going on with them, and sometimes they ask their doctors, but it is them who ask, not the doctors themselves who tell them, and the doctors would confirm that yes, those were sequelae… (Lina, 20, survivor of germ cell tumor)

Another effect of sharing with peer survivors, who have such intense unique experiences in common, enables them to foster their sense of moving on. Thus, even though they have treatment effects that manifest as ailments, the fact of not feeling alone in experiencing them allows survivors to move beyond cancer as the defining feature of their lives. This does not mean that survivors reject their cancer experience altogether, rather, they perceived it was a valuable experience that made them stronger and mainly taught them important lessons about what matters in life—love and relationships.

Once I was asked whether I would change something if I had the chance to live my life again. Honestly, no. It [cancer] is a very hard experience, but it left me with great experiences, great friends, and I think it's thanks to that, that I am who I am now. I like my life how it is now, the changes that the disease brought about [for the family], I think in part they helped us; I keep the beautiful things of what we have to live through. (Sandra, 32, survivor of ALL)

### 3.3 Need for giving back

Many of our respondents, even if they were not involved in survivors' groups, mentioned their wish to give back to others facing cancer as part of the gift of life that they had been granted by surviving. There was a strong sense of responsibility towards helping youngsters who are in a situation similar to theirs and thus make a difference in cancer patients' experience. This *giving back* allows survivors to live a purposeful life.

If I'm studying nursing, it's because first, somehow, I know the needs a patient has, and second, to maybe be able to talk to them a bit about my life, what I went through, because when you are on treatment you only see the sick ones, you never see the survivors, I never ever saw a survivor, until I started to visit [the foundation]. So, I would like them to see that because I've met many people who say that cancer can't be cured and that people with cancer don't survive. (Lina, 20, survivor of germ cell tumor)

I feel that I have that responsibility… I can't not help. This is my cause. Since I got cured, I realized that if I was alive, if I had gotten this second chance, it was for this. For dedicating myself entirely to help those who now are going through the same I did… In my studies, what I want to learn is focused on this cause… I want to share this life view, this passion, this will to soak up every second, to enjoy life… (Ivan, 21, survivor of ALL)

I love to come to the hospital, I love to talk with the parents, I love the feeling of giving. I feel that at the end of the day, one is more blessed than them [those you are trying to help], because one says, ‘Okay, I'm alive, I went through this, and I'm alive’. And back then you would see it [cure] super far away, and to see now that you really are doing something… (Ana, 26, survivor of ALL)

## 4 DISCUSSION

In this research, we show that survivors in Mexico, El Salvador, and Peru have common experiences and stories regarding their discharge from pediatric oncology services, their contact or lack of contact with other childhood cancer survivors, and their wishes for putting their energies at the service of other children who are now going through a cancer experience.

Literature considering aspects related to childhood cancer survivors' transitions from pediatric to adult health care show that, where in place, transition practices are complex and far from fluent, limited by the resources and structure of the health system as much as by knowledge gaps and information practices of the pediatric and adult health care providers.^7^ A transition is considered to be a process in which properly planned multidisciplinary coordination leads to the childhood cancer survivor being transferred to adult healthcare services, but numerous reports show that such a smooth process is not the experience of many survivors.^8^ Additionally, research in the United States and Europe has documented inconsistent follow‐up care for survivors and limited use of cancer‐related adult care as a generalized problem, including lack of engagement on the part of the survivors in their late follow‐up or transition to adult care.^9^, ^10^ Otth et al. consider that loss to follow‐up could be a possible indicator of transition failure.^7^

In Mexico, El Salvador, and Peru, what the young survivors experienced was a discharge from pediatric care, instead of a transition or transfer to adult care. There are no systems in place for adult services to take over the needs of survivors of childhood cancer in a specialized program in the three settings of this study. This may contribute to feelings of insecurity and loneliness, since, as we showed above, the lack of an organized transition contrasts with the security of a trusted physician or health team regularly looking after them under protocol‐guided instructions, as well as contrasting with the sense of belonging and community that both children and families developed over the treatment trajectory. Additionally, they all understood starting in the diagnosis period that their disease was serious and required good treatment adherence and follow‐up.

### 4.1 Losing eligibility for pediatric care

There is a paucity of literature addressing the experience of completing treatment and transitioning to survivorship in childhood cancer from the perspective of the child, adolescent or young adult. Most publications highlight parent experiences of their child's transition, or childhood cancer survivor's reflections of survivorship in general and not specifically about the immediate end of treatment. Release from a close‐surveillant treatment environment may seem initially appealing to the survivors, but their willingness to consult back with the pediatric oncologist shows that they do not have or trust other options when health ailments appear. This is in line with a Swiss study on childhood cancer survivors who ranked post‐treatment visits with a pediatric oncologist as their top option (e.g., compared to a GP), but it must be noted that in this country, survivors are followed by a pediatric oncologist for 10 years after treatment.^11^ Sadak et al. found that survivors in the United States mentioned wanting to be seen consistently by one of their existing oncologists during their transition to adult care or in other words, “someone that goes on that journey with you” (p. 10).^12^ In this regard, a survey among pediatric oncologists in the United States showed that perceived patients' attachment to providers was the most frequently reported barrier for transfer to adult care.^13^ It's been reported that even where a system is in place, survivors' preferences and personal attachment to their physicians play a role in their use of adult health care and engagement with the follow‐up process.^7^ This reflects survivor statements in this study who relied on their pediatric oncologists for healthcare advice despite being discharged from care since they had no survivorship surveillance program to rely on and did not want to initiate care from an adult health care provider with no knowledge of their cancer experience.

### 4.2 Importance of peer survivors

Once again, there have been few recent studies on the experience of childhood cancer survivors relying on peer (other cancer survivors) support. In the United States, Liptak et al. found that a group of adolescent and young adult survivors of a brain tumor, who participated in a social support group (focus on recreation, art, and communication), felt that being with other survivors with a similar medical history was helpful.^14^ They described an atmosphere of acceptance and understanding in the group that was lacking in their day‐to‐day lives. Some survivors also mentioned reflecting on their experiences as having fewer devastating consequences than other survivors. These findings support the experiences of the survivors in this study who described the importance of peer interactions with other survivors.

### 4.3 Giving back

Survivors in this study noted their need to give back by visiting children on treatment for cancer, entering into a health care profession, or volunteering. This is a topic that has not been well investigated and certainly not in low‐ or middle‐income countries. Yet, Molinaro and Fletcher in a study of Canadian childhood cancer survivors found that the survivors had experienced post‐traumatic growth by giving “back to the pediatric cancer community” (p. 272).^15^ The survivors described giving back to organizations that had supported them during treatment and also sharing their experiences with others receiving treatment to encourage them to remain positive. The Latin American survivors in this study reported that giving back was a way to have a purposeful life, which can also be viewed as a positive achievement, particularly in light of the expected late effects of childhood cancer treatment and increased risk of a second malignancy.

### 4.4 Survivorship guidelines

Working groups from Europe and North America have developed clinical practice guidelines for long‐term follow‐up of childhood cancer survivors.^16^ These guidelines are built under the underlying premise that actual follow‐up is possible and in place. Unfortunately, the literature indicates that this is generally true only in high‐income countries where structured long‐term follow‐up for survivors and not necessarily for those who reach adult age is provided, as noted in a study of 15 high‐income countries and three middle‐income countries,^17^ and a survey in the United States that showed that the largest pediatric cancer programs were the ones that offered better established transition to adult care.^12^ Similarly, the need for programs and guidelines for survivors' transition from pediatric to adult health care in high‐income countries has been recognized.^18^ However, major structural challenges for the implementation of such transition processes involve the wide differences between available resources and hospital systems in place, healthcare systems in each country, and childhood cancer policies, among many others.^19^ Even in rich‐resource settings there is a lack of well‐established follow‐up and support systems for childhood cancer survivors.^20^ In a contrasting experience, in Chile, childhood cancer treatment is provided almost entirely under a national program that secures treatment in the major public hospitals and supports rehabilitation and psychosocial needs through the participation of NGOs. This centralized model that sets childhood cancer as a top priority of a national health policy is the basis for a structured and coordinated collaboration that established a comprehensive national follow‐up program with guidelines encompassing, medical, nursing, psychological, social work, occupational therapy, and education areas.^21^

In limited‐resource settings, the lack of well‐coordinated services within healthcare systems in these countries may delay putting in place an effective transition and follow‐up program. Thus, the role of childhood cancer organizations (e.g., NGOs) is essential, since they have shown to be instrumental in providing a safe and familiar environment to launch initiatives for survivors' psychosocial care and other possibilities for a well‐organized follow‐up program. As mentioned above, these organizations offer peer survivors a space to feel supported and to strengthen a sense of belonging after finishing treatment. In general, the involvement of civil society through organized work of NGOs in the improvement of quality care and outcomes in childhood cancer treatment is not only necessary but has been proven to be highly effective.^22^ Initial priorities of such involvement tend to focus on increasing survival. Figures in many LMICs show that it is time for NGOs to help in advancing strategies for the continued care and support of their growing survivor population.

Continued contact among survivors is possible in Mexico and El Salvador, where survivor groups are supported by local childhood cancer foundations. Still, based on the comments of the survivors in this study, the possibility of regular meetings, a broader outreach to participants, and formal programs to cover survivors' needs as a group would be a constructive effort to improve survivor transitions from cancer treatment to life beyond.

## 5 LIMITATIONS

We did not collect data about institutional protocols for transitioning, or possible adult‐pediatric clinic contacts, although we have indications that these services are fairly disconnected.

We have to consider that changes may have been introduced in the hospitals over time (from the time our respondents received treatment and the time they have been interviewed as survivors) regarding how the end of treatment and end of follow‐up are conducted, and how the patients and their families were informed, or the resources or contacts made available to them. However, apart from meetings supported by NGOs and hospital visits, the survivors in this study were not aware of programs or systems to address their care. We cannot generalize our findings across each country or all of Latin America, due to our small sample.

## 6 CONCLUSION

The survivor stories in this study teach us that even if the healthcare systems in these countries are not ready to implement follow‐up and transition programs for childhood cancer survivors, there are resources, mainly the NGOs and the survivors themselves who can initiate supportive systems in the direction of formal future strategies. Initiatives mobilized at a basic level, as presented above, address what many survivors want and need to do as part of their after‐treatment life. That is, survivors giving to and receiving peer support from fellow survivors, which is not only beneficial but also crucial for their well‐being. These initiatives can then be leveraged to create long‐term programs that are inclusive of the specific survivor needs in each setting and to eventually reach a level of multi‐institutional programs. Institutional coordination between existing NGO foundations and hospitals is possible and should be encouraged as a next step in the process of building a robust program.^23^

Most survivors of childhood cancer in Latin America will eventually adapt and cope, but we do not know how well since we have no published data. Our interviews with survivors in Mexico, El Salvador, and Peru have shown that they experience a new level of unexpected challenges, although many have found ways to cope through giving back and peer‐to‐peer support. Therefore, it appears that a follow‐up program is essential, and aligns with the right of the survivors to receive transitional support from pediatric oncology programs before final discharge to help them manage the consequences of their cancer experience. Cancer, at least for the survivors and their families, does not end the day of the patient's last chemotherapy. Latin American pediatric oncology programs would benefit from querying their survivors and NGOs to learn about transitions and build locally appropriate, and culturally acceptable survivor surveillance programs in concert with said NGOs to maximize the potential synergy of efforts on behalf of this growing and important survivor population.

## CONFLICT OF INTEREST

The authors have no conflict of interest to declare.

## AUTHOR CONTRIBUTIONS

All authors had full access to the data in the study and take responsibility for the integrity of the data and the accuracy of the data analysis. *Conceptualization*, N.R., M.F.O.‐S., J.C.; *Methodology*, N.R., M.F.O.‐S., J.C.; *Investigation*, M.O.S. has to be included together with N.R; *Writing—Original Draft*, N.R., J.C.; *Writing—Review & Editing*, N.R., M.F.O.‐S., J.C.; *Data Curation*, N.R.

## ETHICAL STATEMENT

Appropriate permission to conduct the studies was arranged according to local practice in each country. Permission to conduct the study was granted by two relevant hospitals, one in Peru and one in El Salvador, and by the Mexican NGO, because the children had been treated in multiple hospitals. Participants subsequently gave explicit oral consent for participation in the research before the start of the interview. All names used in this article are pseudonyms.

## Data Availability

Research data are not shared.
